# Assessment of retinal vein pulsation through video-ophthalmoscopy and simultaneous biosignals acquisition

**DOI:** 10.1364/BOE.486052

**Published:** 2023-05-12

**Authors:** Radim Kolar, Tomas Vicar, Jiri Chmelik, Roman Jakubicek, Jan Odstrcilik, Eva Valterova, Michal Nohel, Karolina Skorkovska, Ralf P. Tornow

**Affiliations:** 1 Department of Biomedical Engineering, Faculty of Electrical Engineering and Communication, Brno University of Technology, Brno, Czech Republic; 2 Deparment of Ophthalmology and Optometry, St. Ann University Hospital, Brno, Czech Republic; 3 Department of Ophthalmology and Optometry, Masaryk University, Brno, Czech Republic; 4 Department of Ophthalmology, Friedrich-Alexander-University Erlangen–Nürnberg, Erlangen, Germany

## Abstract

The phenomenon of retinal vein pulsation is still not a deeply understood topic in retinal hemodynamics. In this paper, we present a novel hardware solution for recording retinal video sequences and physiological signals using synchronized acquisition, we apply the photoplethysmographic principle for the semi-automatic processing of retinal video sequences and we analyse the timing of the vein collapse within the cardiac cycle using of an electrocardiographic signal (ECG). We measured the left eyes of healthy subjects and determined the phases of vein collapse within the cardiac cycle using a principle of photoplethysmography and a semi-automatic image processing approach. We found that the time to vein collapse (T_vc_) is between 60 ms and 220 ms after the R-wave of the ECG signal, which corresponds to 6% to 28% of the cardiac cycle. We found no correlation between T_vc_ and the duration of the cardiac cycle and only a weak correlation between T_vc_ and age (0.37, p = 0.20), and T_vc_ and systolic blood pressure (-0.33, p = 0.25). The Tvc values are comparable to those of previously published papers and can contribute to the studies that analyze vein pulsations.

## 1. Introduction

The retina is the only human tissue that allows direct non-invasive imaging of the microvascular circulation. Therefore, pulsatile changes induced by the cardiac cycle can be observed throughout the retina, mainly at the optic nerve head (ONH) and in the peripapillary region. These pulsatile phenomena include venous pulsation (often referred to as spontaneous venous pulsation, SVP), arterial pulsation (including changes in artery diameter and bending), and changes in light absorption of a specific wavelength due to changes at the microcapillary and vascular level (i.e. retinal photoplethysmography, PPG) [1,2]. The pulsating nature of these phenomena is primarily driven by the cardiac cycle. However, other factors also contribute and influence retinal pulsations. Since the intraocular space is close to the brain and the dura of the brain extends as a sheath around the ONH, cerebrospinal fluid pressure (CSFP) influences some of these retinal phenomena. Another variable is intraocular pressure (IOP), which can primarily influence SVP and PPG pulsations. Both pressures (CSFP and IOP) are not constant but also pulsate (see, e.g., IOP pulsations [3] or CSFP pulsations [4]).

In this paper, we focus on the measurement of vein pulsation and its analysis. These pulsations are often referred to as spontaneous vein pulsations, although they are not actually **“**spontaneous**”** because they are controlled by temporal changes in the translaminar pressure gradient [5]. Therefore, the heart is the **“**pulse generator**”**, and the magnitude and phase of IOP and CSFP create the condition for the vein to collapse. Another factor that can influence SVP is venous capacitance [5], which defines the stretching ability of the vein wall. The pressure gradient is most evident at the ONH, and therefore SVPs are observed primarily in this region. Since the translaminar pressure gradient is the result of the difference between IOP and CSFP, which are influenced by the cardiac cycle, it can be assumed that SVPs are also dependent on the cardiac cycle. This has been confirmed by several studies [6–14]. However, there is one question that remains open: what is the timing or phase of SVP during the cardiac cycle? Some studies have been published on this topic [7–9,11,15], but the results are not uniform. The importance of this parameter could arise when considering ocular diseases associated with changes in blood flow (e.g. retinal vein occlusion, glaucoma) or when considering cardiovascular diseases such as carotid stenosis, as the latter affects retinal blood flow [16]. Furthermore, SVP can be used to estimate intracranial pressure [17], where the SVP phase may also play a significant role. Finally, an accurate measurement of the SVP phase could help to better understand its origin and possible future applications.

In SVP analysis, an appropriate spatial and temporal resolution must be used. Spatial resolution is usually not a problem because some commercially available retinal imaging solutions are used. However, the temporal resolution should be high enough to capture potential variability within the subject and between subjects in the SVP phase. Several studies published on this topic have revealed important information about SVP [5,15,18–21], but many of them use a relatively small temporal sampling rate (typically around 10 fps). Some studies have used a retinal vascular analyzer (RVA, Imedos UG, Jena, Germany), which allows the acquisition of retinal image sequences with a sufficient frame rate (around 25 fps), but the light intensity is relatively high, which is not comfortable for subjects.

Here, we present a novel hardware solution for acquiring retinal video sequences and physiological signals using trigger pulses that preserve the possibility of synchronous analysis. The advantage of this solution is that it is relatively easy to setup and use in a clinical setting. In addition, it uses low-intensity pupil illumination to make the measurement more comfortable for the subject. Last but not least, it works with both dilated and non-dilated pupils. Furthermore, we utilize a photoplethysmographic principle to analyze the pulsation, which does not need an exact extraction of the vein diameter. We tested our setup in a group of healthy subjects and analyzed the SVP phase during the cardiac cycle. Finally, the results are discussed and compared with other published work on SVP analysis.

## 2. Materials and methods

### 2.1 Acquisition system

**Fig. 1.**
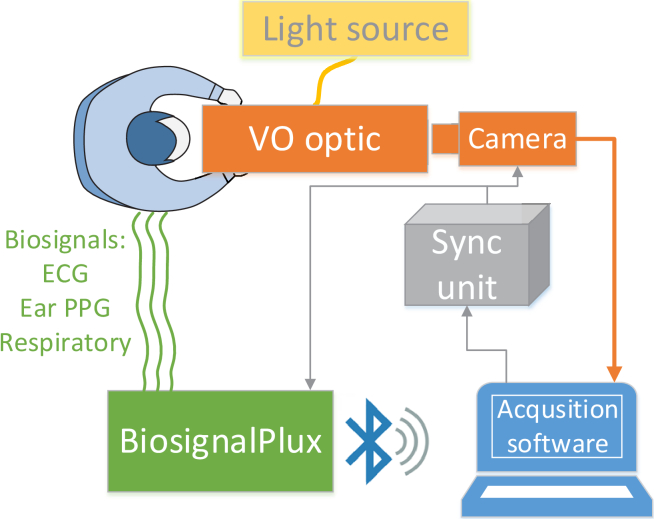
Illustration of our proposed acquisition setup.

The acquisition system consists of three main parts (Fig. 1):

**1. Video-ophthalmoscope (VO)** – The monocular video-ophthalmoscope is based on our previous research on binocular video-ophthalmoscopy [1,2]. The setup of the version used in the previous work was modified to a monocular version with the possibility of multispectral measurement [22]. This device acquires retinal video of the optic nerve head and the peripapillary area with a field of view of 20° × 17° (1224 × 970 pixels). This corresponds to an approximate area of 6 × 5 mm in the retina for a subject with an axial length of 24 mm. The frame rate of the CMOS camera (UI-3060 Rev 2, USB 3.0, IDS Imaging Development Systems GmbH, Germany) was set at 25 fps (camera exposure time approximately 40 ms) and the light power generated by an external light source (CoolLED pe-4000, CoolLED Ltd., UK) in the plane of the eye pupil was less than 15 µW. This results in a retinal illumination of 50 µW/cm^2^, which corresponds to 16.6% of the maximum exposure limit according to [23]. The lengths of the video sequence are between 10 and 15 seconds.
**2. Biosignal acquisition unit** - The BiosignalPlux 4 channel unit (PLUX Wireless Biosignals S.A., Lisbon, Portugal) was used in this setup. We used an electrocardiographic (ECG) sensor, an ear photoplethysmography (PPG) sensor, and a respiratory band to record the corresponding signals. In this study, we used only the ECG signal. The fourth channel was used to record the trigger pulses (see the next paragraph). All signals were sampled at a sampling rate of 1000 Hz.
**3. Synchronization unit** - This unit consists of an Arduino platform which generates a square trigger signal to drive the camera acquisition. Each rising edge of this square signal triggers the frame acquisition. At the same time, this trigger signal is recorded by the BiosignalPlux unit together with the measured biosignals. This enables us to precisely determine the time of each frame within the biosignals. Thus, the retinal video and biosignals are precisely synchronized.

### 2.2 Subjects

Thirteen healthy subjects were measured after pupil dilation (tropicamide drops, concentration of 0.25%). First, blood pressure (Omron, Intellisense) and intraocular pressure (Huwitz, non-contact tonometer HNT-1) were measured. The fundus image with a clinical fundus camera (Canon CR-1 with Canon EOS 40D). During measurement with a video-ophthalmoscope, we used various wavelengths [22], but here we analyzed video sequences, which were acquired with 525 nm. Light with this wavelength provides a good contrast of the blood vessels.

This study followed the principles of the Declaration of Helsinki for research involving human subjects, and informed consent was obtained from all study participants. The measurement procedure was reviewed and approved by the Ethics Committee of Brno University of Technology (no. EK:03b/2021).

### 2.3 Signal and image processing

The ECG signal was processed to detect the positions of the R-wave in each cardiac cycle. We proposed an automatic detection that consists of filtering and robust local maxima detection. The signal was filtered with a median filter (size 10), high-frequency components were removed with a Gaussian filter (σ=12), and the baseline was removed by subtraction of a low-frequency signal obtained with a Gaussian filter (σ=200). Then, the robust local maxima detector with a peak threshold, minimal peak distance, and minimal peak prominence (minimal value drop between peaks) was used to detect the positions of R-waves. Here, the parameters were set adaptively (i.e. automatically) for each signal – threshold as (signal maximum) ⁄ 4, peak prominence as (signal maximum – signal minimum) ⁄ 2 and minimal distance as 0.545 seconds (maximum 110 beats per minute). This leads to the correct detection of all R-waves in all signals, which was confirmed by visual inspection as well.

Similarly, the rising edges of the acquired synchronization square trigger signal were detected with the same local maxima detector, where the detector was applied to the first-order difference of this signal; however, detection without filtering and peak prominence was sufficient. A threshold half of the square wave magnitude was applied with a minimal peak distance of 0.6×square wave period.

The simultaneous acquisition of biosignals and video allows us to determine the temporal position of each R-wave in video recording. Thus, the frame that corresponds to the position of the R-wave can be determined. It should be noted that due to the different sampling rates for biosignals and video sequences, the exposure time of each frame (40 ms) corresponds to 40 samples in the ECG signal.

**Fig. 2.**
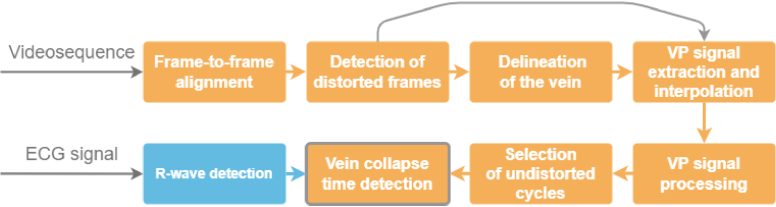
The main flow diagram of the methodology; VP refers to vein pulsation.

Retinal image sequences were processed in several steps corresponding to partial blocks in Fig. 2 (see the flow diagram in Fig. 2):

**Frame-to-frame alignment** – The previously published approach [22] was used to align each frame with respect to the first frame (i.e. reference). This approach corrects for translational and rotational movements using a combination of phase correlation and tracking of the center lines of the blood vessels. The registration error of this approach is 0.78 ± 0.67 pixels inside the optic disc and 1.39 ± 0.63 pixels outside the optic disc, respectively [22].

**Detection of distorted frames** - Due to the eye movements, which may also occur during the frame exposure time, some frames can be blurred. Furthermore, blinking artifacts also cause significant distortion. These distorted frames cannot be restored and must be detected before analysis. Our previously developed method [24] for different data sets did not provide satisfactory results for these new sequences. Therefore, a new approach was used based on the similarity of the frames with their neighbors was used; specifically, the Euclidean distance (ED) between each frame and the median image from 71 surrounding frames was calculated and used for the detection of distorted frames. From EDs, distorted frames were detected as outliers using the generalized extreme Studentized deviate test [25], where most extreme values of ED are iteratively removed until outliers are not recognized with the test. This unsupervised automatic detection was refined manually by visual inspection of frames with higher ED values compared to their neighbors, which can contain weak distortion.

**Vein delineation** - In the next step, only the pulsating part of the central retinal vein was manually delineated in ImageJ. A freehand tool was used to create one region on the vein during video playback.

**Fig. 3.**
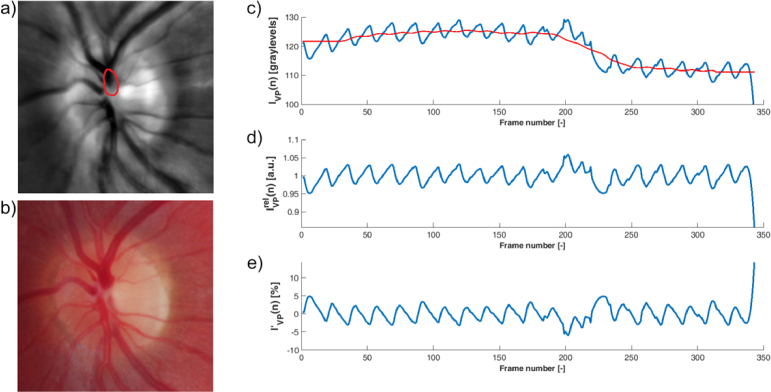
a) An average image of a sequence with manually delineated ROI for vein pulsation analysis. b) Corresponding color fundus image. c) Vein pulsation signal extracted as an average value from ROI from each frame. The red curve represents the *moving average* signal, *I_AVG_(n)* d) Relative pulsatile signal, $I_{VP}^{rel}(n)$ . e) Photoplethysmographic representation of vein pulsatile signal, $I_{VP}^{,}(n)$ .

**Vein pulsation signal extraction** - The region of interest (ROI) from the previous step was then used to extract the average intensity from this ROI in each frame. This results in a signal representing the pulsation of the vein (referred to here as the VP signal, denoted I_VP_(n)). In this signal, each cardiac cycle can be clearly recognized (Fig. 3). The minimum in each cycle represents the lowest intensity in ROI, which corresponds to the maximum diameter of the vein (the vein creates a dark structure inside the ONH). In contrast, the maximum value for each cardiac cycle represents the collapse of the vein, because during this collapse, the diameter of the vein decreases (see Fig. 3).

**SVP signal interpolation** – The indexes of the corrupted frames (Step 2) were used for interpolation of the corresponding values of VP signal from their neighbors (1D cubic interpolation).

**SVP signal processing** – Reflected intensity from the retina can also be distorted by illumination intensity changes (caused mainly by small eye and head movements and the fluctuation of the pupil diameter), see Fig. 3(c). This affects especially the low-frequency part of the VP signal. To compensate for these changes, we computed the relative intensity $I_{VP}^{rel}(n)$ as [2]:

$$I_{VP}^{rel}(n)=\frac{I_{VP}^{}(n)}{I_{AVG}^{}(n)},$$

where *I_AVG_(n)* represents a low-frequency component estimated by a moving average filter with the length of the window equal to the 1.5 multiple of the average cardiac cycle length. The typical length of the impulse response was around N = 35 samples. This relative signal (see the example in Fig. 3(d)) fluctuates around 1 and allows us to easily define an alternative representation of vein pulsation as

$$I_{VP}^{,}(n)\;[\%]=100(1-I_{VP}^{rel}(n))=100\left(1-\frac{I_{VP}^{}(n)}{I_{AVG}^{}(n)}\right).$$

This representation can be interpreted as a photoplethysmographic signal, where the value is related to the volume of the corresponding part of the vein. Due to the minus sign in the equation, we now have a signal, where each minimum represents the minimum volume (i.e. vein collapse) and each maximum is the maximum vein volume during the cardiac cycle. An example of this signal is shown in Fig. 3(e).

**Selection of undistorted cardiac cycles** – Even though we detected the distorted frames and used the interpolation, there can still be some parts of the VP signal, where the particular cardiac cycles are distorted. This is mainly due to the blinking of the eyes or large movements of the eye. These distorted cycles were manually excluded from the analysis. Finally, the number of undistorted cardiac cycles for each subject was typically between 5 and 11 cycles.

**Fig. 4.**
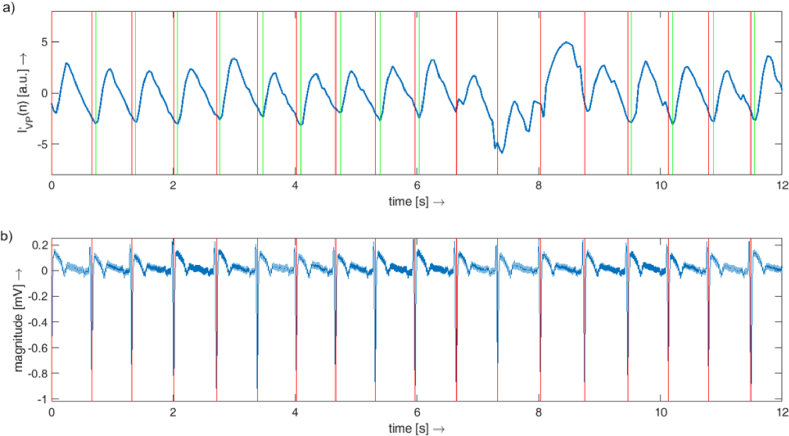
a) Vein pulsation signal. b) ECG signal. The red vertical lines show the position of detected R-waves. The green vertical lines represent the time of the vein collapses. A small variation of the interval between the red and green lines can be observed.

**Detection of vein collapse** - Vein collapse is represented by a local minimum during each cardiac cycle in the VP signal. The position of each minimum was detected in a fixed temporal window (220 ms) after the R-wave. Visual inspection of each detected minima showed the correctness of each position. The length of the window was set according to an assumption based on the literature review (see Discussion). The plot of the simultaneously acquired ECG and VP signal is shown in Fig. 4, together with the detected R-waves and vein collapses.

## 3. Results

### 3.1 Correlation among clinical parameters and vein collapse time

**Fig. 5.**
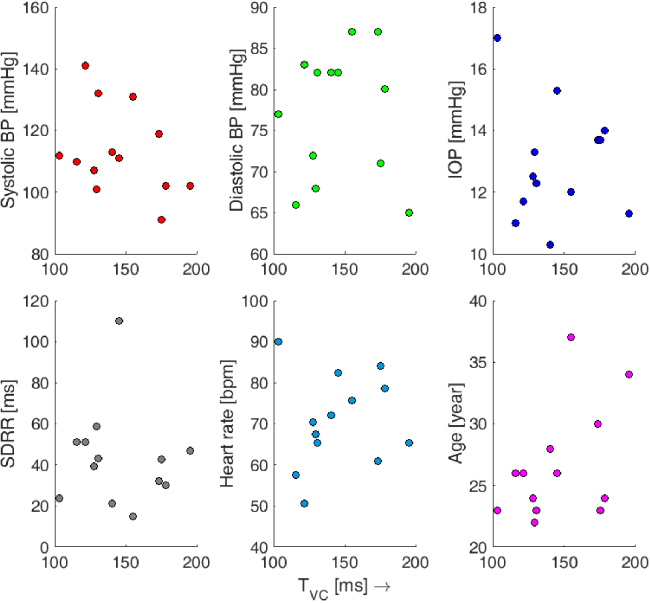
Scatter plots between vein collapse time (T_vc_) on the x-axis and particular parameters on the y-axis: blood pressure (BP; systolic and diastolic), intraocular pressure (IOP), standard deviation of RR intervals (SDRR)**,** heart rate and age.

Scatter plots between T_vc_ and other parameters are plotted (Fig. 5) and the correlation was evaluated using the Spearman correlation coefficient. We found a significantly higher correlation coefficient only for systolic BP (-0.33, p = 0.25) and age (0.37, p = 0.20). The remaining calculated Spearman correlation coefficients were below 0.2.

**Fig. 6.**
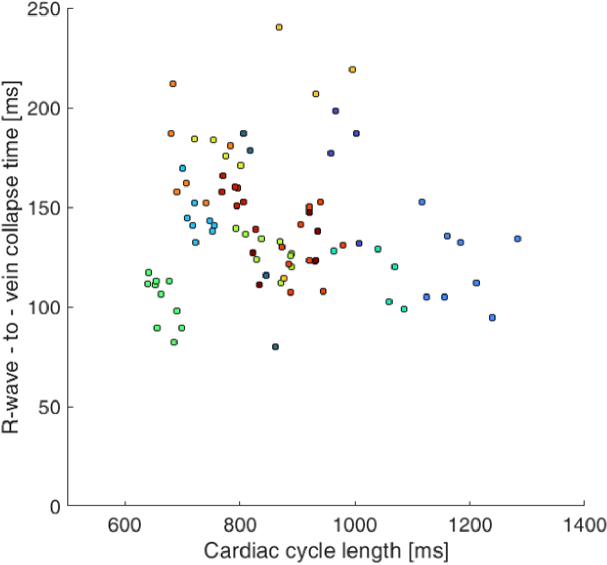
The scatterplot of the TVC collapse time and the cardiac cycle length. Each color represents one of 13 subjects.

Another not well-known fact is the relation between T_vc_ and the duration of the cardiac cycle. The scatter plot in Fig. 6 shows this relation, where each dot represents single cardiac cycle, and each colour represents one subject (in total 85 cardiac cycles from all 13 subjects were considered). The Spearman correlation coefficient was below 0.1, showing that these values are not correlated.

### 3.2 Vein collapse time analysis

**Fig. 7.**
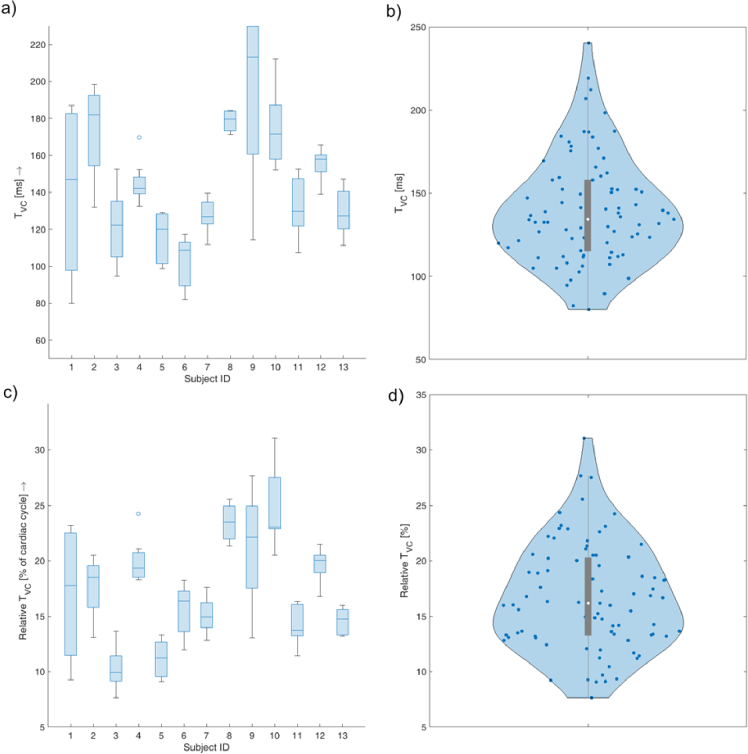
a) Boxplot of T_VC_ for each subject. b) Violin plot of T_VC_ for all subjects. c) Boxplot of *relative* T_VC_ for each subject. d) Violin plot of *relative* T_VC_ for all subjects.

The T_vc_ was determined for each undistorted cardiac cycle (according to the VP signal). These values were visualized as a boxplot for each subject and as a one violin plot for all subjects together (Fig. 7). Similarly, T_vc_ was expressed in milliseconds and in percent with respect to the duration of the cardiac cycle.

**Fig. 8.**
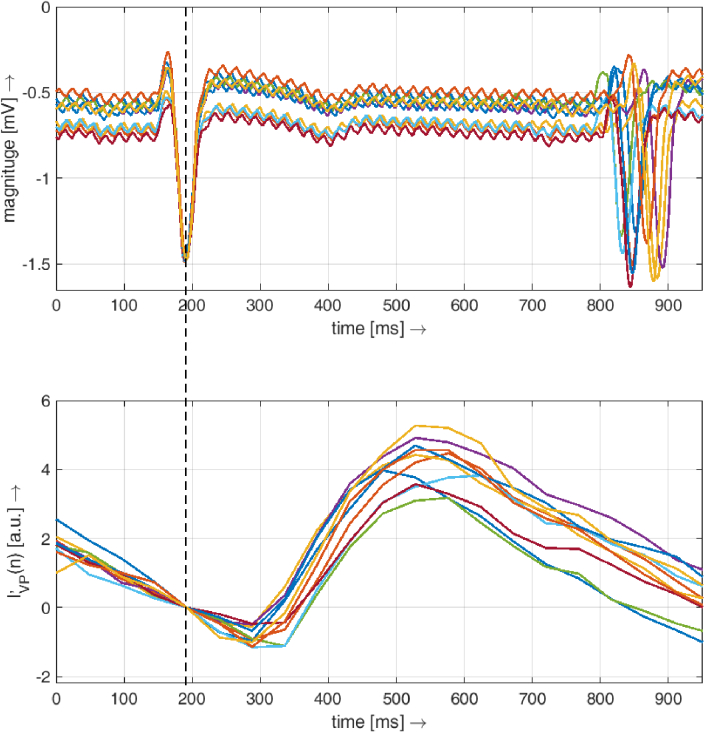
An example of several cardiac cycles represented by the ECG signal (a) and the VP signal (b) of one subject. The heart rate variability is clearly visible as different lengths of each heartbeat in the ECG signal. The vertical dashed line represents the time of the R-wave. The VP signals for each cardiac cycle were vertically aligned to zero at the time of the peak of the R wave.

Our setup and analysis also allow us to plot a single cardiac cycle represented by the VP signal and the ECG signal. This representation clearly shows the natural heart rate variability of the subject during the acquisition; see an example for one subject in Fig. 8. However, as shown above, no correlation was found between T_vc_ and SDRR (representing this heart rate variability).

## 4. Discussion

**Fig. 9.**
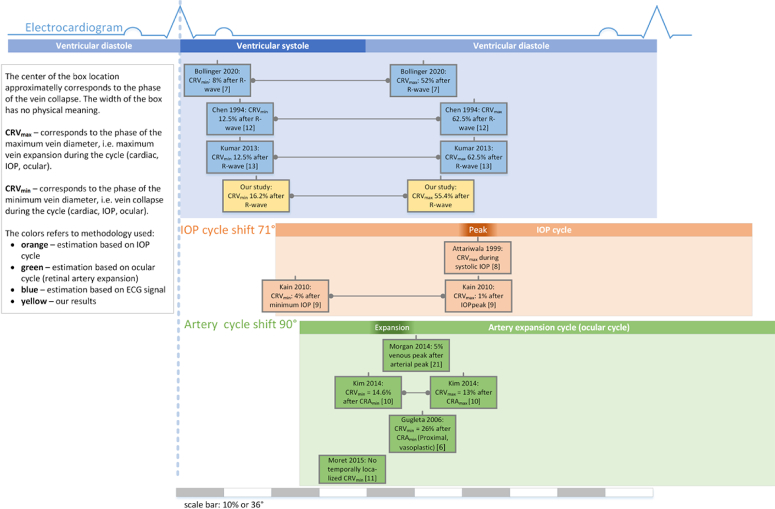
A graphical summary of published results analyzing vein pulsation and comparison with our results. The blue part contains our results (yellow boxes) and other results that used ECG to analyze VP. The orange part contains papers using IOP pulsation, and green part summarizes papers utilizing retinal artery pulsation.

Our study investigates vein pulsation and vein collapse phase in relation to the cardiac cycle, represented by the ECG signal in healthy subjects and using the photoplethysmographic principle. The results clearly show (Fig. 7) that vein collapse occurs in a systolic phase of the cardiac cycle in healthy subjects. To compare our findings and get a more complex view, including also the IOP cycle and the retinal artery cycle, we graphically summarize this in Fig. 9.

We did not measure IOP pulsation; however, this IOP ocular cycle begins later than the cardiac cycle due to the time needed to induce changes in IOP after cardiac contraction and aortic valve. It was reported [25] that IOP cycle begins at 71° of the ECG cycle and peaks at 220°. The relationship between retinal arterial expansion and ECG can also be found in the same study [26], where the flow cycle in the central retinal artery begins at 46° and peaks at 111° of the cardiac cycle. The same comparison approach was also used by Levin et al. [5]. The relation between the cardiac cycle, IOP cycle and artery expansion cycle (also referred to as the ocular circulatory cycle) is shown in Fig. 9 as blue, orange and green horizontal bars, respectively. This enables us to compare our results with more published results using IOP or artery expansion, not only with ECG signals.

The boxes in Fig. 9 represent the central retinal vein maximum diameter phase (CRV_max_) or central retinal vein minimum diameter phase (CRV_max_) published by various authors. These phases are typically evaluated in the studies and are expressed as a percentage of the cardiac cycle (i.e. range 0–100%) or in degree (i.e. 0–360°). Thus, CRV_min_ represents a phase of vein collapse because the vein shrinks when it collapses. CRV_max_ then represents the phase of the maximum diameter of the change in the vein, which occurs during the translaminar pressure gradient (decrease). The horizontal central position of each box in Fig. 9 is given by the average values (i.e. phase) of CRV_max_ and CRV_min_, respectively, which were published in respective papers. The widths of the boxes have no physical meaning.

The results of the proposed approach show that the estimated Tvc values are in agreement with the results published in the papers, which use the ECG signal as a *trigger* (the blue part in Fig. 9) [7,12,13]. Shortly, Chen et al. [12], in 1994, used ECG to trigger fundus camera acquisition in 8 increasing delays after the R-wave. Thus, the temporal resolution was only 1/8 of the cardiac cycle, i.e. 125 ms if we consider 60 heartbeats per minute. The authors manually evaluated the diameter of the vein according to the intensity profile in 10 healthy subjects. Almost twenty years later, Kumar et al. [13] used the same principle of data acquisition (with the same temporal resolution), but utilized a more advanced image processing approach for the estimation of vessel diameter to eliminate the subjectivity provided by manual evaluation. They evaluated 12 healthy subjects. Bollinger et al. [7] in 2020 took advantage of a commercially available RVA device (Imedos UG, Jena, Germany) and connected this retinal camera with an ECG device for simultaneous acquisition. The diameter of the vessel was also extracted using the RVA software. Subsequently, they also measured the IOP curve simultaneously with the phases of the ECG to determine the IOP during the cardiac cycle. They measured 21 healthy subjects, but finally used only 11 recordings due to poor image quality.

Given all these results, we see that the phase of vein collapse and expansion is not dependent on the hardware used for data acquisition. Similarly, it does not depend on the image processing approach - we used a photoplethysmographic approach that does not need vein diameter extraction, as all the previous studies, which used vein diameter measurement. This can simplify the (possibly automated) analysis because only vein segmentation within the ONH would be required for this VP analysis.

The comparison in Fig. 9 also shows that vein pulsations are in phase with IOP and retinal arterial pulsation. This is, of course, dependent on the IOP phase shift values and the values of the arterial pulsations shift relative to systole, given by the ECG signal. Here, we have used approximate values that are probably not the same in all individuals. It is also clear that agreement with studies which utilized IOP or the ocular circulatory cycle is weaker (see the reddish and green boxes in Fig. 9). This can be caused by many factors. The IOP cycle and arterial cycle delay with respect to the R-wave do not need to be the same for each subject, and, of course, the precision of these delays is limited. Here we used delays, which were inferred from a study by Harazny et al. [26] The recent study by Bollinger et al. [7] showed that the delay between the R-wave and IOP beginning is at 21° and the peak is at 165°. This is an almost 50 ° difference from the values in Harazny et al. If we consider this difference, then we would get more comparable results with IOP-based studies based on IOP [8,9].

Furthermore, some studies used very small frame rate for retinal video acquisition (9 fps by Moret et al. [11] or 8 fps by Kim et al. [15]), which results in a temporal resolution greater than 100 ms. This limits the precision of the results achieved because the sampling interval cannot reliably capture the dynamic of the vessel pulsation. Finally, hardware used in some studies can also limit the precision of vein collapse time detection, e.g. Attariwala et al. [8] used scanning laser ophthalmoscope recorded with the S-VHS recorder, Kain et al. [9] used *an audible beep* signal produced by an oximeter and microphone to trigger the recordings.

However, it should be noted that the variability in vein collapse time is relatively large between subjects. In our study, we found values ranging from 60 to 220 ms, which corresponds to 6% to 28% of the cardiac cycle. Similar variability can also be found in other papers, e.g. Kain et al. [9] reported a time delay from minimum IOP to vein collapse between –23 ms to 107 ms; Kim et al. [10] reported vein collapse in 14.8%±5.1% of the ocular circulatory cycle. Furthermore, we also showed that there are subjects with relatively stable T_vc_ (subjects 4, 7, 8, 12) or rather unstable T_vc_ (subjects 1, 9). Thus, we can speculate that this *vein collapse variability time* could also be an indicator of higher temporal fluctuation of the translaminar pressure gradient. However, we observed that even with high heart rate variability (Fig. 8), the duration of the link between the cardiac cycle and the vein collapse time is probably negligible (Fig. 6).

Regarding the correlation between T_vc_ and other parameters, we found a higher Spearman correlation for systolic BP (-0.33, p = 0.25) and age (0.37, p = 0.20). Although the p-values are relatively high, we should be aware of the possible effect of these variables in future studies. However, more subjects are needed to confirm this weak correlation. However, in our recent paper, we found that age can modulate the morphology of the VP curve [27]. Another study [28], which supports this hypothesis, showed that the occurrence of SVP was significantly associated with systolic blood pressure, suggesting *a relationship* between VP and systolic BP.

The VP is often used as a screening tool to rule out elevated ICP. Wong et al. [29] reported high sensitivity (0.89) of the VP to exclude increased ICP, but low specificity (0.15) and low agreement (influencing the sensitivity and specificity). Therefore, an objective automated SVP evaluation would help to make this ICP analysis more robust. Simultaneous acquisition of retinal video and ECG signal can help significantly in this task, because the time interval of possible vein pulsation can be easily defined by detecting the R-wave and thus can increase the reliability of this approach.

Further research will focus on extending image analysis to the retinal arteries and also on infrared imaging with wavelengths greater than 780 nm. The application of this wavelength range would increase the comfort of the subject being examined. However, the contrast of blood vessels is usually much lower with a flood-illuminated camera due to lower light attenuation [30]. This will probably affect the image analysis part, because the magnitude of the pulsation signal will be smaller. Thus deeper experimental analysis and evaluation is needed.

## 5. Conclusion

This study introduced a novel hardware solution to measure retinal videos and biosignals simultaneously. The setup of the video-ophthalmoscope is relatively easy to build and enables high flexibility to be adjusted for a specific application. We introduced a semi-automatic approach for processing the acquired data based on photoplethysmography and we demonstrated the usability of this approach for VP analysis. The results we achieved are comparable to those of already published papers and can contribute to studies analyzing vein pulsations in the retina.

## Data Availability

Data are available on request from the corresponding author.
